# Training basic laparoscopic skills using a custom-made video game

**DOI:** 10.1007/s40037-013-0106-8

**Published:** 2014-01-10

**Authors:** Jetse Goris, Maarten B. Jalink, Henk O. ten Cate Hoedemaker

**Affiliations:** Department of Surgery and Post Graduate School of Medicine (Wenckebach Institute), University Medical Center of Groningen, Huispostcode FC20, Postbus 30.001, 9700 RB Groningen, the Netherlands

**Keywords:** Game, Laparoscopy, Wii, Skills, Training

## Abstract

Video games are accepted and used for a wide variety of applications. In the medical world, research on the positive effects of playing games on basic laparoscopic skills is rapidly increasing. Although these benefits have been proven several times, no institution actually uses video games for surgical training. This Short Communication describes some of the theoretical backgrounds, development and underlying educational foundations of a specifically designed video game and custom-made hardware that takes advantage of the positive effects of games on basic laparoscopic skills.

## Background

The use of video games for training basic laparoscopic skills, such as inverted movements, eye–hand coordination, depth perception, and ambidexterity, is not an entirely new concept. In the past ten years, the combination of games and surgery has gained much interest, and the scientific literature on the subject is rapidly increasing. It is considered proven that video game experience correlates with laparoscopic skills [1, 2], and a number of controlled experiments have shown that video games can be used to increase basic laparoscopic skills in novices in the short- and middle-long term.

Schlickum et al. [3] showed that a group of medical students, after 5 weeks of training with a first person shooter for at least half an hour a day for 5 days a week, scored significantly better on a laparoscopic simulator than a matched group that had not been playing video games at all. Similarly, a short and temporary warm-up effect on basic laparoscopic skills was observed in several studies using a three-dimensional balance game for a game console or a mobile phone [4, 5].

Although the positive effects of video games on the aforementioned skills can be considered legitimate, no actual game has been developed to benefit from these positive effects. One article written by Bokhari et al. [6] in 2010, however, describes how a custom-made controller add-on for the motion sensitive Wii console (Nintendo Co., Ltd., Kyoto, Japan) can be used to play an existing balance game. Using this add-on, the Wii Remote mimics a laparoscopic instrument and can be used to train eye-hand coordination. A group of surgical residents, who completed 50 different levels using this method, were significantly faster, better and moved more proficiently with an electrocautery procedure on a virtual reality simulator than the control group.

Cutting Edge, a collaboration between the University Medical Center Groningen, game developer Grendel Games and the LIMIS Institute for Healthcare Innovations and Training, is creating a video game specifically aimed at laparoscopic training. By developing both advanced Wii Remote add-ons and custom video game software, we hope to create a method that is fun, cost-effective and able to help surgical novices train basic laparoscopic skills better than traditional games. Underground will differ from existing video games in the sense that the movements made during the game will have a lot more in common with laparoscopy than traditional games, and it is our theory that it can be validated in the same manner as virtual reality surgical simulators.

## The game

**Fig. 1:**
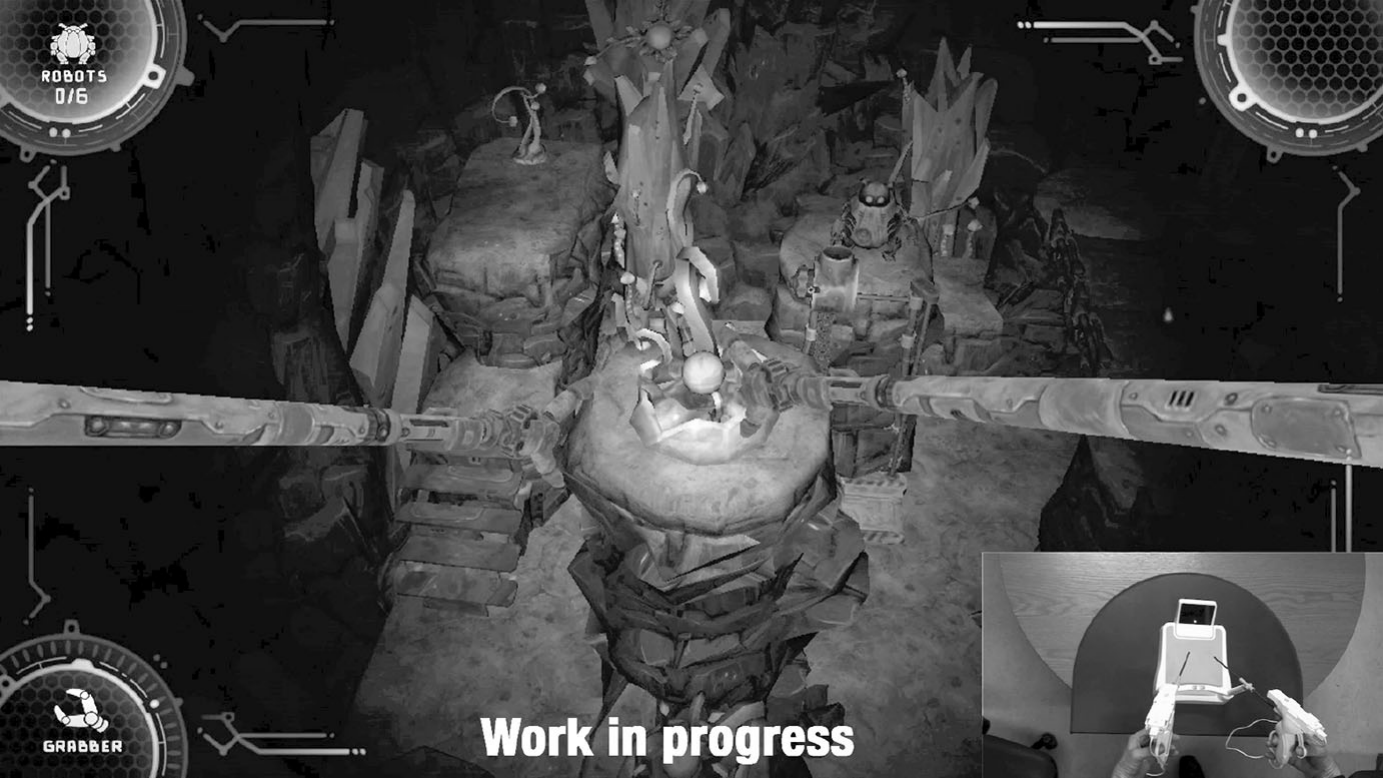
Screenshot of the game and the custom controller

In Underground (working title), players use two Wii Remote controllers in custom-made laparoscopic tool shells to play a game that is based on movements made during laparoscopic surgery. In contrast to simulators, the game does not contain actual medical content, but comprises a story-driven mode, based on a fictional world where the player has to help small robots to escape from a mine. To aid the robots in their escape, the player controls two large robotic arms and demolishes and rebuilds the environment of the mine (Fig. 1).

The concept of a mine was chosen because laparoscopic surgeons also work in a primarily dark area and have to break things (adhesiolysis, ligation of mesentery, resections) before they can start to rebuild (anastomoses, hernia repairs). In the process, the development of a traditional simulator was knowingly avoided.

The gameplay of Underground consists of a mixture of action and puzzles in a sandbox-like environment. The main goal, escaping from the underground world, can be reached by solving puzzles that require basic laparoscopic skills, cognitive skills and problem-solving skills.

During the game, the players learn to handle analogous versions of laparoscopic equipment under familiar circumstances such as the lack of depth perception and inverse movements. The game has four distinct themes, each with five different levels and a final boss. To defeat the boss at the end of each world, the player has to combine all the skills that were learned during the previous levels.

To ensure that the learning goals (inverted movements, eye–hand coordination, depth perception, and ambidexterity) are met in the game, the game-based activities were aligned with the learning goal activities, using the constrained design approach to instructional computer games, as described by Shelton and Scoresby [7]. One learning goal, for example, is the training of depth perception. The associated game-based activities are freeing frozen robots from ice by touching them, killing enemies by using the electric wielder tool and picking up scrap metal in a three-dimensional world. In another example, ambidexterity is trained by collecting scrap metal by holding it with one instrument and drilling it loose with the other instrument.

The player is rewarded for handling the equipment carefully and minimizing collateral damage. Causing collateral damage results in the crumbling of walls or falling stalactites, impeding progress to the exit of the level for some robots. The less collateral damage you cause, the more robots you save.

## Validation

Although Underground is not an actual simulator, it will be validated through a traditional validation process, as described by the European Association of Endoscopic Surgeons in their consensus guidelines for the validation of virtual reality surgical simulators [8]. In this process, called test validation, face, content, construct and concurrent validity of a simulator need to be obtained before one can state that such a machine actually measures or tests what it is supposed to: basic laparoscopic skills.

A prototype of the game, which contained a single level aimed at training all four learning goals, has proven to possess a solid construct and concurrent validity [9]. This was achieved by comparing the scores of experts and novices (surgeons and internists, respectively) on both the game and a gold standard test. Not only is there a significant difference in score between experts and novices, but a high correlation between both the video game and gold standard scores was found as well.

Face validity of a near-final version of the game was obtained at the Chirurgendagen 2013, a three-day annual congress of the Netherlands Society of Surgery, by letting experts play the game and letting them fill in a questionnaire on the hardware and software, based on similar research in the field. The results are currently being analyzed and will be submitted for publication shortly.

Finally, content validity, which deals with the correctness of medical content in a surgical simulator, cannot be tested in Underground (or any serious game for that matter) because the game completely lacks medical content, such as anatomy or procedural knowledge, on purpose. The authors will therefore refrain from testing this validity.

Once the test validation on the final version of the game is finished, we can start looking at the actual effects of the game on basic laparoscopic skills, the so-called experimental validation. As noted before, video games can positively influence basic laparoscopic skills in both the long and short term [3–5]. Whether Underground possesses similar training or warming-up effects will be studied in controlled experiments using interns and residents when the game is ready for release.

## Conclusion

Games could be used to train basic laparoscopic skills in surgical trainees, but to our knowledge no actual institution does so. Therefore, we are developing a motion-sensitive video game that uses custom-made Wii Remote add-ons that is specifically aimed at training those skills, hoping to be of better use than normal games and creating a fun and affordable training method. While the video game hardware and software is currently still under development, we have already started a traditional validation process, proving that our game has acceptable face, construct and concurrent validity in comparison to existing simulators. However, more research on both the test and experimental validity of the game should be carried out before we can actually use it in surgical training. The Wii U video game and its hardware are aimed for release at the end of 2013.

## References

[1] Badurdeen S, Abdul-Samad O, Story G, Wilson C, Down S, Harris A (2010). Nintendo Wii video-gaming ability predicts laparoscopic skill. Surg Endosc.

[2] Rosser JC, Lynch PJ, Cuddihy L, Gentile DA, Klonsky J, Merrell R (2007). The impact of video games on training surgeons in the 21st century. Arch Surg.

[3] Schlickum MK, Hedman L, Enochsson L, Kjellin A, Felländer-Tsai L (2009). Systematic video game training in surgical novices improves performance in virtual reality endoscopic surgical simulators: a prospective randomized study. World J Surg.

[4] Sadandanan S, Dryfhout VL, Sosnowski JP (2008). Video games and laparoscopic surgery. J Gynecol Surg.

[5] Plerhoples TA, Zak Y, Hernandez-Boussard T, Lau J (2011). Another use of the mobile device: warm-up for laparoscopic surgery. J Surg Res.

[6] Bokhari R, Bollman-McGregor J, Kahoi K, Smith M, Feinstein A, Ferrara J (2010). Design, development, and validation of a take-home simulator for fundamental laparoscopic skills: using Nintendo Wii for surgical training. Am Surg.

[7] Shelton BE, Scoresby J (2011). Aligning game activity with educational goals: following a constrained design approach to instructional computer games. Educ Technol Res Dev.

[8] Carter FJ, Schijven MP, Aggarwal R (2005). Consensus guidelines for validation of virtual reality surgical simulators. Surg Endosc.

[9] Jalink MB, Goris J, Heineman E, Pierie JPEN, ten Cate Hoedemaker HO. Construct and concurrent validity of a Nintendo Wii video game made for training basic laparoscopic skills. Surg Endosc. 2013 Sep 6. 10.1007/s00464-013-3199-6 24061627

